# NK cells adjuvant therapy shows survival benefits in a gastric mixed signet ring cell carcinoma patient: A case report

**DOI:** 10.1097/MD.0000000000024979

**Published:** 2021-03-12

**Authors:** Yuan-Yuan Jin, Wen-Zhuo Yang, Zheng-Yang Sun, Zhong-Bo Wang, Jian Chen, Chun-Tao Wu, Zhao-Yong Yang

**Affiliations:** a NHC Key Laboratory of Biotechnology of Antibiotics, Institute of Medicinal Biotechnology, Chinese Academy of Medical Sciences, Beijing; b Sun Yat-sen University School of Medicine, Guangzhou; c Department of Oncology, The Affiliated Yantai Yuhuangding Hospital of Qingdao University, Yantai, Shandong; d North China University of Science and Technology Affiliated Hospital, Tangshan, China.

**Keywords:** chemotherapy, distal gastrectomy, gastric signet ring cell carcinoma, natural killer cells adjuvant therapy, tumor markers

## Abstract

**Rationale:**

Advanced signet ring cell (SRC) carcinoma has a worse prognosis. Therefore, early diagnosis and prevention is particularly important; SRC tumors have lower R0 resection rate and are thought to be less chemosensitive than non-SRCC. Consequently, a novel postoperative adjuvant treatment is urgently needed to improve clinical outcomes.

**Patient concerns:**

A 41-year-old female with advanced gastric SRC carcinoma was treated with radical gastrectomy and oxaliplatin-based regimen for 6 cycles after surgery. She was suspected of recurrence with the high level of carbohydrate antigen (CA) 72-4.

**Diagnoses:**

The gastroscopy revealed SRC carcinoma of gastric antrum and poorly differentiated adenocarcinoma in some areas. The diagnosis of postoperative pathology report was gastric cancer with stage III C (T4a, N3a, M0).

**Interventions:**

The level of CA72-4 rapidly increased during the 2 follow-up after the completion of conventional treatment, ex vivo-cultured allogeneic natural killer (NK) cell infusion was offered to prevent recurrence.

**Outcomes:**

Intravenous injections of NK cells combination with surgical treatment and chemotherapy showed therapeutic effects in this patient with possible relapse. The patient remained disease-free 46 months after the infusion of NK cells until the latest follow-up.

**Lessons:**

CA72-4 appeared to be the most sensitive and specific marker in the gastric cancer patient, and the high level of CA72-4 may indicate the risk of recurrence. This case report provide rationale for NK cell infusion following the rapid increase of CA72-4 to prevent recurrence.

## 1 Introduction

Gastric cancer (GC) is one of the most common malignancies worldwide, particularly in East Asia, East Europe, and South America.^[1]^ Surgery is still the main treatment for operable GC. Postoperative chemotherapy is a recommended component of resectable GC therapy and has been shown to improve patient outcome in several studies. However, 30% to 50% of patients relapse within 5 years after surgery and adjuvant chemotherapy.^[2,3]^ Signet-ring cell (SRC) carcinoma, an unfavorable subtype of GC is a unique name for its particular histological features and clinical behavior. If treated early, signet ring cell carcinoma has a better prognosis than other subtypes; however, advanced signet ring cell carcinoma has a prognosis that is even worse than undifferentiated adenocarcinoma.^[4]^

Early diagnosis of signet ring cell carcinoma is, therefore, critical to guide optimal treatment, but the typical presentation during conventional endoscopy is a pale, flat lesion, especially difficult to realize the early detection, so there is limitation to the early diagnosis, judgment of reoccurrence and evaluation of efficacy for tumors. The detection of tumor markers could reflect the occurrence and development of tumors timely and the detection process is convenient and rapid, with high sensitivity. Therefore, they have been the important clinical means of auxiliary examination in the clinical diagnosis and prognosis evaluation for tumors.^[5,6]^ Among the available tumor markers, carcino-embryonic antigen (CEA), carbohydrate antigen (CA) 19-9, and CA72-4 are widely used in the follow-up of patients with gastrointestinal malignancies. These markers have been demonstrated to be useful in the diagnosis, treatment, and prognosis of GC.^[7,8]^

The potential of natural killer (NK) cells in comparison with other immune cells to induce cytotoxicity in the absence of prior sensitization caused them to be an attractive candidate for immunotherapy. Given the difficulties of sourcing abundant numbers of cytotoxic NK cells from peripheral blood, additional strategies have been investigated to provide readily available banks of NK cells for patients. Umbilical cord blood (UCB) is an amazing source of NK cells for immunotherapy. The merits of UCB derived NK cells including availability of UCB units, high percentage of NK cell progenitors, collecting different source of UCB together, lower risk of graft versus host disease, and less stringent requirements for human lymphocyte antigen matching making UCB an off the shelf source for NK cells immunotherapy.^[9]^

In view of this, we developed ex vivo expansion techniques that can induce cord blood mononuclear cells to directly differentiate into high cytotoxic NK cells using a cocktail of cytokines and interleukin-2. The culture method of high cytotoxic NK cells will be introduced in another article. Here, we report a case of gastric mixed signet ring cell carcinoma, who received radical gastrectomy for GC and oxaliplatin-based chemotherapy before using our NK cells immunotherapy. In this study, we focus on the survival benefits of NK cells immunotherapy and clinical utility of tumor markers CEA, alpha-fetoprotein, CA15-3, CA12-5, CA19-9, CA72-4, human chorionic gonadotropin, neuron-specific enolase, and potential blood biomarkers of T-cell subsets in monitoring response and predicting the prognosis of the patient.

## 2 Case presentation

**Figure 1:**
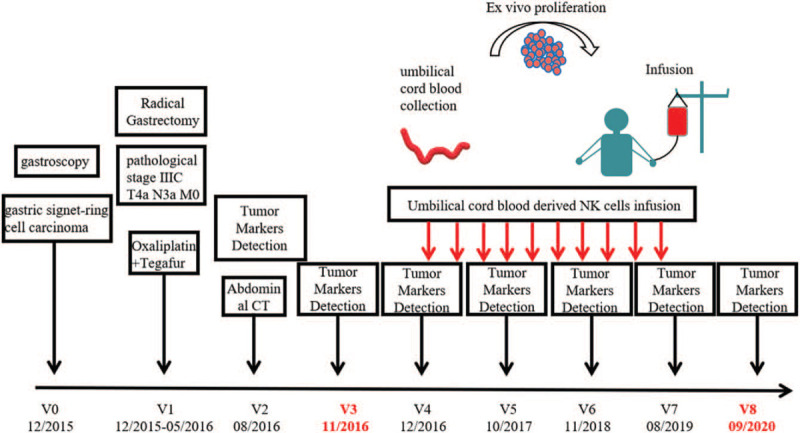
Schematic representation of the clinical history, therapy, and visits of a patient diagnosed with gastric signet-ring cell carcinoma (stage III C, T4a, N3a, M0) in December, 2015 (V0). The patient was treated with oxaliplatin and tegafur combined chemotherapy for 6 cycles after the radical gastrectomy during the period of December 2015 to May 2016 (V1). The level of CA72-4 decreased significantly after the treatment (V2). Three months later (V3), the level of CA72-4 increased to 86.34 U/mL rapidly. On November, 2016 (V4), the patient began to receive NK cell treatment on a 3-yearly basis. The detection of tumor markers and the abdominal CT scan after NK therapy (V4–V8) revealed that it was becoming normal and in good condition. CA = carbohydrate antigen, CT = computed tomography, NK = natural killer.

**Figure 2:**
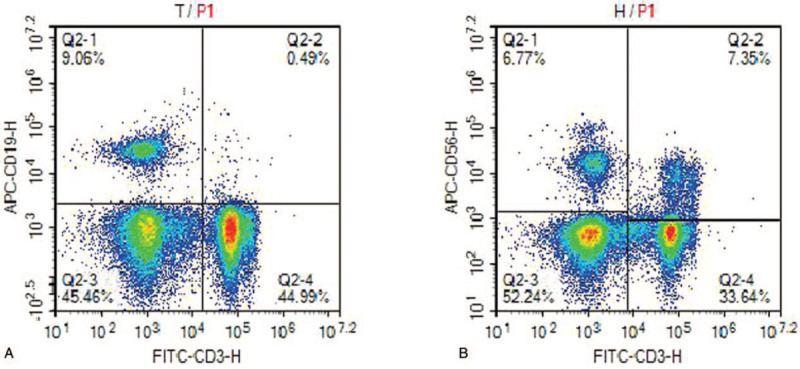
Dot plots of percentages of CD3 + T and NK cells in peripheral blood of the patient by flow cytometry analysis. NK = natural killer.

We report the case of a 41-year-old female. The patient presented paroxysmal vomiting for more than 1 year. She underwent gastroscope evaluation and was diagnosed with gastric SRC carcinoma at hospital. Six days later, she was admitted to the other hospital and completed the preoperative examination. On December 30, 2015, she underwent radical (R0) gastrectomy under general anesthesia. The diagnosis of postoperative pathology report was as follows: ulcerative poorly differentiated adenocarcinoma in the lesser curvature of gastric antrum, most of them were signet ring cell carcinoma, only a small portion were mucinous adenocarcinoma, the tumor size was 7∗6∗1.8 cm, invading the full thickness of gastric wall, and the tumor thrombus can be seen in the vascular, no cancer tissue was found in the surgical margin of the upper and lower, but partial lymph node metastasis in the greater curvature of stomach. Immunohistochemically, the tumor showed positivity for cytokeratin, and the Ki-67 labeling index was 5%. Staining for human epidermal growth factor receptor-2 and human epidermal growth factor receptor-1 was negative. On the basis of the above findings, the tumor was diagnosed as GC with stage III C (T4a, N3a, M0). The patient was treated with oxaliplatin and tegafur combined chemotherapy for 6 cycles after the operation. The last chemotherapy time was on May 18, 2016. Eight months after the operation the patient stayed well and free for recurrent disease. Serum samples for CEA, alpha-fetoprotein, CA15-3, CA12-5, CA19-9, CA72-4, human chorionic gonadotropin, and neuron-specific enolase levels were measured during chemotherapy, and only the levels of CEA and CA72-4 were above the cut-off levels (Table 1). With the progression of chemotherapy, the level of CA72-4 decreased significantly (Table 1). However, the level of CA72-4 began to increase rapidly at the second follow-up after chemotherapy on September 20, 2016 (Table 1). Meanwhile, we designed to investigate the percentages of CD3+T, CD3+CD4+T, CD3+CD8+T, B, and NK cells in peripheral blood of the patient using a single-platform flow cytometry-based method, and to analyze the immune function of the patient when the tumor marker CA72-4 increased to the level of 86.34 U/mL. As a result, a lower percentage of CD3+T and NK cells were observed (Fig. 2), it stated that the patient’ immune function was impaired. For fear of recurrence and metastasis after chemotherapy with the impaired immune function, the patient began to receive NK cells immunotherapy on November, 2016, using ex vivo-generated NK cells from UCB, at a dose of 2 × 10^9^ CD56^+^/CD3^–^ cells, intravenously, 3 times a year, up to September 2020. In the subsequent follow-up after NK cells immunotherapy, the level of CA72-4 decreased rapidly, as of the latest follow-up, the level of CA72-4 had dropped to normal (Table 1) and she was observed to be in a very good condition, without evidence of disease progression (Fig. 1).

**Table 1:** Serum levels of CEA, AFP, CA15-3, CA12-5, CA19-9, CA72-4, HCG-BET, and NSE at different time points.

Time	CEA ng/mL	AFP ng/mL	CA15-3 U/mL	CA12-5 U/mL	CA19-9 U/mL	CA72-4 U/mL	HCG-BET mIU/mL	NSE ug/L
March 31, 2016	3.06	1.57	11.74	14.32	24.10	35.26↑	0.10	10.52
May 17, 2016	4.22↑	1.59	10.70	12.99	27.49	18.24↑	0.10	12.12
August 11, 2016	4.34↑	1.17	8.97	16.40	16.02	12.37↑	0.10	10.39
September 20, 2016	2.62	–	–	–	–	46.67↑	–	–
November 22, 2016	3.17	–	–	–	–	86.34↑	–	–
November 30, 2016	2.10	1.67	7.64	20.60	14.87	16.68↑	0.10	8.27
December 23, 2016	0.676	1.73	9.06	27.13	15.14	11.31↑	0.10	13.31
February 10, 2017	–	–	–	–	–	12.82↑	–	
October 18, 2017	–	–	–	–	–	7.06↑	–	–
November 6, 2018	2.84	1.48	8/39	13.12	17.42	6.68	0.889	9.79
August 8, 2019	0.882	1.66	6.76	11.10	9.02	9.23↑	0/901	8.57
September 8, 2020	0.91	1.10	6.89	9.68	8.73	3.82	1.06	8.64

AFP = alpha-fetoprotein, CA = carbohydrate antigen, CEA= carcino-embryonic antigen, HCG-BET = human chorionic gonadotropin, NSE= neuron-specific enolase.

## 3 Discussion

CA72-4 was first described by Colcher et al in 1981.^[10]^ It is a glycoprotein with a molecular mass >1000 kDa and is a tumor marker for numerous cancers, including breast, ovarian, colorectal, and pancreatic cancer, and it has good specificity for GC.^[11,12]^ Although a large number of previous studies have shown that CEA, CA19-9, CA72-4, CA12-5 can be used as gastrointestinal tumor markers, the study of GC shows that CA72-4 has high sensitivity and specificity.^[13,14]^ The analysis showed that CA72-4 was the preferable single test, with a sensitivity value (93.83%) that was higher than that of CEA (72.20%) and much higher than that of CA19-9 (22.30%).^[15]^ In the literature, CA72-4 was associated with advanced tumor stage, lymph node metastasis, and distant metastasis.^[16–22]^ Compared with other indicators, the increase of CA72-4 in follow-up patients often precedes the clinical detection of tumor recurrence.^[23]^ In addition, CA72-4 was significantly correlated with tumor cell proliferation.^[24]^ Ychou et al showed that the survival time of GC patients with abnormal serum CA72-4 was significantly lower than that of normal patients. In this study, CA72-4 appeared to be also the most sensitive and specific marker in the GC patient. At the beginning of treatment, the level of CA72-4 decreased from 35.26 U/mL to 18.24 U/mL, which can be seen as a response to treatment. However, the level of CA72-4 increased from 12.37 U/mL to 86.34 U/mL during the 3 follow-ups after chemotherapy. Because of the rapid increase of CA72-4 levels, we are worried about the risk of recurrence and metastasis despite the abdominal computed tomography scan showed no abnormality.

On November 2016, the patient began to receive NK cells immunotherapy. In our study, we improved the preparation method of NK cells. Our technique resulted in a high yield of at least 1.0 × 10^10^ NK cells. These NK cells showed evident cytotoxicity against GC cell lines in vitro. One week after the NK cells infusion, the level of CA72-4 dropped to 16.68 U/mL (Table 1). For the next 3 years, the patient continued to receive NK cells immunotherapy. In the lately follow-up, the level of CA72-4 had dropped to the normal and the abdominal computed tomography scan also found no abnormality.

Currently, surgical resection is still the primary treatment for many solid malignancies, however, scattered tumor cells that remain after resection of the primary tumor, may be the main trigger factor for disease recurrence.^[25]^ Furthermore, surgical procedures may induce the release of immunosuppressing factors that render host immune surveillance ineffective, ultimately leading to the increased metastatic disease or recurrence following surgery.^[26]^ NK cells are cytotoxic lymphocytes that constitute a major component of the innate immune system. NK cell dysfunction following surgery has been documented in both human patients^[27–29]^ and animal models.^[30,31]^ Rate of local recurrence following surgical tumor resection of colorectal cancer correlated with lower NK cell levels.^[32]^ Correlations between reduced NK cytotoxicity and incidence of metastasis have been established in head and neck as well as pharyngeal cancer.^[33–35]^ Circulating NK cells had a prognostic role in OS in advanced GC.^[36]^ These examples highlight the potential for NK cell immunotherapies to improve patient outcomes.^[37]^

Traditional postoperative adjuvant radio/chemotherapy may eliminate residual lesions and reduce tumor recurrence to some extent, but, pure adjuvant radio/chemotherapy primarily kills actively proliferating tumor cells rather than relatively indolent cancer stem cells, which are mainly responsible for recurrence.^[38,39]^ In addition, SRCC is thought to be less chemosensitive than non-SRCC. Therefore, a novel postoperative adjuvant treatment is urgently needed to improve clinical outcomes for these patients. Pay an attention to the immune state of patients through detection of the level of lymphocyte subsets including percentage and number timely and accurately, it will help us to evaluate conditions of prognosis and adjust the treatment program for patients. Although there is untapped potential in the use of immunotherapies to reverse or prevent surgical stress-induced NK cell dysfunction. But for the critical role in the anti-tumor immune response of NK cells, we adopt the way of infusion the expansion of NK cells in vitro to repair the damaged immune function and prevent the recurrence and metastasis.

## 4 Conclusion

In conclusion, this is the first demonstration of NK cells adjuvant therapy in combination with surgical treatment and chemotherapy on in patient with advanced gastric SRC carcinoma. The treatment decisions were fully compliant with the patient’ choices, better reflecting practical clinical situations. NK cells infusion combined with surgical and chemotherapy was well tolerated and showed great potential for the prevention of gastric SRC carcinoma recurrence and prolonging of survival.

## Author contributions

**Data curation:** Wen-Zhuo Yang.

**Methodology:** Zheng-Yang Sun, Zhong-Bo Wang.

**Resources:** Chun-Tao Wu.

**Writing – original draft:** Yuan-Yuan Jin.

**Writing – review & editing:** Jian Chen, Zhao-Yong Yang.
